# Congenital toxoplasmosis among hospitalized infants in Poland in the years 2007–2021: study based on the national hospital registry

**DOI:** 10.1038/s41598-023-38270-y

**Published:** 2023-07-08

**Authors:** Michał Rząd, Krzysztof Kanecki, Katarzyna Lewtak, Paweł Goryński, Piotr Tyszko, Izabela Lewandowska-Andruszuk, Aneta Nitsch-Osuch

**Affiliations:** 1 Department of Social Medicine and Public Health, Medical University of Warsaw, 3 Oczki Str., 02-007 Warsaw, Poland; 2 Doctoral School, Medical University of Warsaw, Warsaw, Poland; 3 National Institute of Public Health NIH - National Research Institute, Warsaw, Poland; 4 Institute of Rural Health in Lublin, Lublin, Poland; 5 Department of Obstetrics, Gynaecology and Gynaecologic Oncology, Mazovian Specialist Hospital, Radom, Poland; 6 Faculty of Medical Sciences and Health Sciences, Kazimierz Pulaski University of Technology and Humanities, Radom, Poland

**Keywords:** Paediatrics, Epidemiology

## Abstract

Congenital toxoplasmosis (CT) is a rare entity and it may pose a life-threatening risk for the newborns. The aim of the study was to evaluate the incidence and other selected factors of CT in Poland. Our study is a population-based study on CT patients in 2007–2021. The study was based on 1504 hospitalization records of first-time diagnosis of CT in newborns. In the study group, we observed 763 males (50.7%) and 741 females (49.3%). The mean and median age was 31 days and 10 days, respectively. Based on the hospital registry, the mean annual CT incidence was estimated to be 2.6 per 10,000 live births (95% CI 2.0–3.2 per 10,000 live births). The incidence of CT cases fluctuated over the years 2007–2021, with the highest incidence in 2010 and the lowest one in 2014. There were no statistically significant differences between the incidence of CT in relation to sex or place of residence. The periodic fluctuations in the number of cases of congenital toxoplasmosis indicates the need to develop effective prevention programs to effectively counteract the disease and its consequences.

## Introduction

*Toxoplasma gondii* infection is one of the most common protozoal infections in the human population^1^. *T. gondii* causes a disease in humans called toxoplasmosis. The main reservoir of *T. gondii* in the environment is found in rodents, which can then be ingested by cats, which are the only known definitive host of this parasite. Humans can become infected by ingesting food or water contaminated with cat faeces, eating raw or undercooked meat, through blood transfusions, during the solid organ transplantation or from the mother through the placenta^1,2^. A special form of toxoplasmosis is congenital toxoplasmosis (CT). It is defined as a vertical infection of the foetus and it takes different clinical forms. The severity of the disease depends on the gestational age at which the mother became infected. After maternal infection in the last trimester of pregnancy, infants usually do not present symptoms at birth, while those infected in the first or second trimester are at an increased risk of serious complications such as miscarriage, intrauterine growth restriction, intrauterine calcification, hydrocephalus, retinitis and other ocular and neurological diseases^3,4^. In the first trimester, the risk of disease transmission is the lowest, of a few to 15%, in the second trimester it is just over 40%, and in the third trimester it rises to over 70%^5^. In contrast, the symptoms of CT are found more often in the case of an infection in the early stages of pregnancy. On average, the absolute risk of vertical transmission to the foetus is about 25–29%. Postnatally, only 10% of foetuses show clinical signs. One-third of the diseases have a severe course^6^. As pregnancy progresses, the risk of foetal intracranial lesions from CT decreases^7^. Another issue is the increased rate of miscarriages and intrauterine foetal deaths in the case of infections at an early stage of pregnancy^8^. The diagnosis of *T. gondii* infection in pregnancy is important for the prevention of the complications that this infection can cause in the foetus and later in the newborn. CT can cause permanent neurological and ophthalmic damage^9,10^. In the diagnosis of toxoplasmosis in a pregnant woman, serological response has a significant importance. During the diagnostic procedure, the titres of antibodies produced after contact with the protozoan at different stages of pregnancy are evaluated. If an active infection is detected, targeted treatment is possible^10,11^. What is more, with prenatal ultrasound, congenital malformations in the foetus can be visualized^10^.

In Germany, current data show a high rate of past *Toxoplasma gondii* infections in the general population, ranging from 20 to 77%, depending on age^12^. In Turkey, the incidence rate of toxoplasmosis ranges from 17.5 to 69.5%^11^.

In France, 0.2–0.25% of women become infected with *T. gondii* during pregnancy^6^. In a study of TORCH antibody seroepidemiology in China, the overall prevalence of IgG anti-*T. gondii* in women of childbearing age was 1.71% and IgM antibodies were 0.30%. Moreover, the rate of primary *T. gondii* infections in the study population was 0.08%^13^. Another study based on TORCH screening in 18,104 women at Xi'an University Hospital found *T. gondii* IgG/IgM seropositivity of 4.35%/0.35%^14^.

The global estimated incidence of CT varies by region from 5 to 34 cases per 10,000 live births and it averages 15 cases per 10,000 live births worldwide^15^. Detailed epidemiological studies from recent years indicate that the CT incidence varies in different regions of the world, which may be related to access to medical services in a given country and socio-economic conditions. The following incidence rates have been observed across countries: 1 per 10,000 live births in Austria, 2.1 per 10,000 in Denmark, 2.9 per 10,000 in France^16–18^. Studies from Morocco have shown an incidence of 3.9–8 per 10,000^19^. Data from Brazil vary from study to study and show the incidence of 4–23 per 10,000^7,20,21^. There are no current epidemiological data from the US, and the most recent ones estimate the incidence of CT at 1 per 10,000^21^. A cross-sectional study conducted in China found CT incidence of 0.07 per 10,000 hospitalized newborns in 27 children’s hospitals in the years 2015–2020^22^. The rate is as high as 18 per 10,000 in Panama, Latin America, where the history of *T. gondii* infection can be found in more than 90% of the population at the age of over 60^23^.

Data from Poland are limited. In Poland, the standard of perinatal care is defined by the decree of the Polish Minister of Health from August 16, 2018. One of the pillars of this legislation are guidelines for the prevention of infectious diseases in the perinatal period. According to the guidelines, every pregnant woman should have a titre of IgM and IgG antibodies to *T. gondii* measured by the 10th week of pregnancy, and in the case of a negative IgG result, the test should be repeated between the 21st and 26th week of pregnancy^24^. There are no data that assess CT incidence at the national level. Available studies refer to single obstetric and neonatal centres, or they are limited to single regions of the country. The median age of women diagnosed with primary toxoplasmosis during pregnancy was 28 years^25^. The incidence of CT in Poland, based on data from the Poznan region in 1998–2000, was determined at 10.8 per 10,000^26^. In a study conducted in 8281 pregnant women in Lodz in the years 2004–2012, IgM and IgG antibodies to T. gondii were detected in 9.7% and 40.6% of patients, respectively. Based on literature data on the transmission rate in pregnancy, the incidence of CT was estimated by the authors at 18 per 10,000^27^.

The importance of an early diagnosis and treatment of CT for public health may constitute a crucial argument for the need to undertake research in the field of CT; in particular, the prevalence of this disease in Poland and changes in its occurrence in recent years.

The aim of the study was to evaluate CT incidence and its trends in recent years in Poland, and present comorbidities and other factors related to this disease (including the place of residence: rural versus urban areas).

## Methods

Our study is a population-based, retrospective analysis of hospital discharge records of infants with CT. Data were obtained from the National Institute of Public Health in Poland, and they covered the period from 2007 to 2021. All hospitals in Poland, except psychiatric facilities, are legally required to send discharge data to the Institute. The data are anonymous and include information on hospitalizations with ICD-10 code diagnoses, dates of admission and discharge, sex, date of birth, and place of residence. All hospitalization records with primary or secondary ICD-10 P37.1 code diagnosis were included in the study. In order to limit the impact of secondary hospitalizations on the incidence of CT, the authors excluded repeated hospitalizations. A retrospective study was performed on all patients who were hospitalized in the years 2007–2021. Information on the study was submitted to the local bioethics committee. CT often requires advanced differential diagnostic procedures and treatment during hospitalization. Therefore, hospitalized cases may provide a good basis for estimating the incidence. We assumed that CT diagnoses were made in hospitals on the basis of the most current and widely used diagnostic criteria.

Based on ICD-10 codes, we analysed associated diseases in the study group. Diseases were analysed within larger subject groups of ICD-10 that ranged from A00 to U89. R00-R99 ICD10-codes were not taken into consideration. Due to the inclusion criteria and the presence of code P37.1 in all patients, the analysis of comorbidities in the P00-P99 range was refined to specific ICD-10 codes. We presented the most frequent diseases that occurred in a minimum of 5% of patients in the analysis.

To perform most statistical analyses, Statistica (TIBCO Software Inc, version 13) was used^28^. WINPEPI^29^ was used to perform chi-square tests. For continuous variables with normal or non-normal distribution, respectively, means and 95% confidence intervals or medians and IQR were computed. For nominal variables, counts and percentages were analysed. Hospitalization rates related to CT infection were calculated as the estimated number of unique patients per 10,000 live births, using data (national census) from the Central Statistical Office of Poland^30^. To assess trends, we used linear regression. To assess normal distribution, we used Shapiro–Wilk test. We applied the Levene’s test to evaluate equality of variances. When normality assumptions were not met, non-parametric tests (Chi-square, U Mann–Whitney) were applied. A two-sided p-value lesser than 0.05 was considered to be statistically significant.

### Ethics approval and consent to participate

This study did not involve human participants, data, or tissue. It was conducted using only aggregated and anonymized data. Institutional review board approval was not required. All methods were carried out in accordance with relevant guidelines and regulations.

## Results

**Figure 1:**
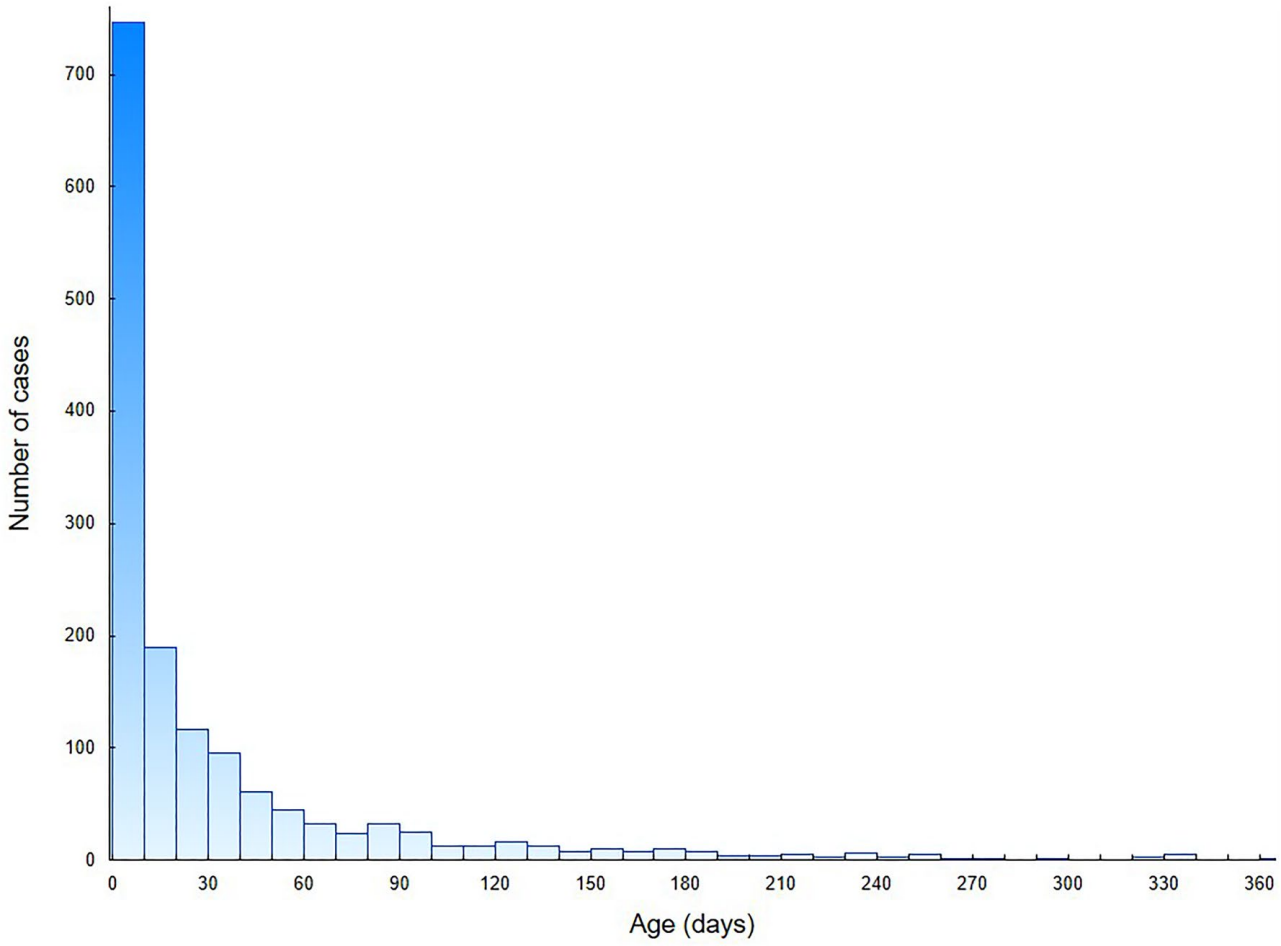
Number of first-time CT hospitalizations in Poland per patients' age.

**Figure 2:**
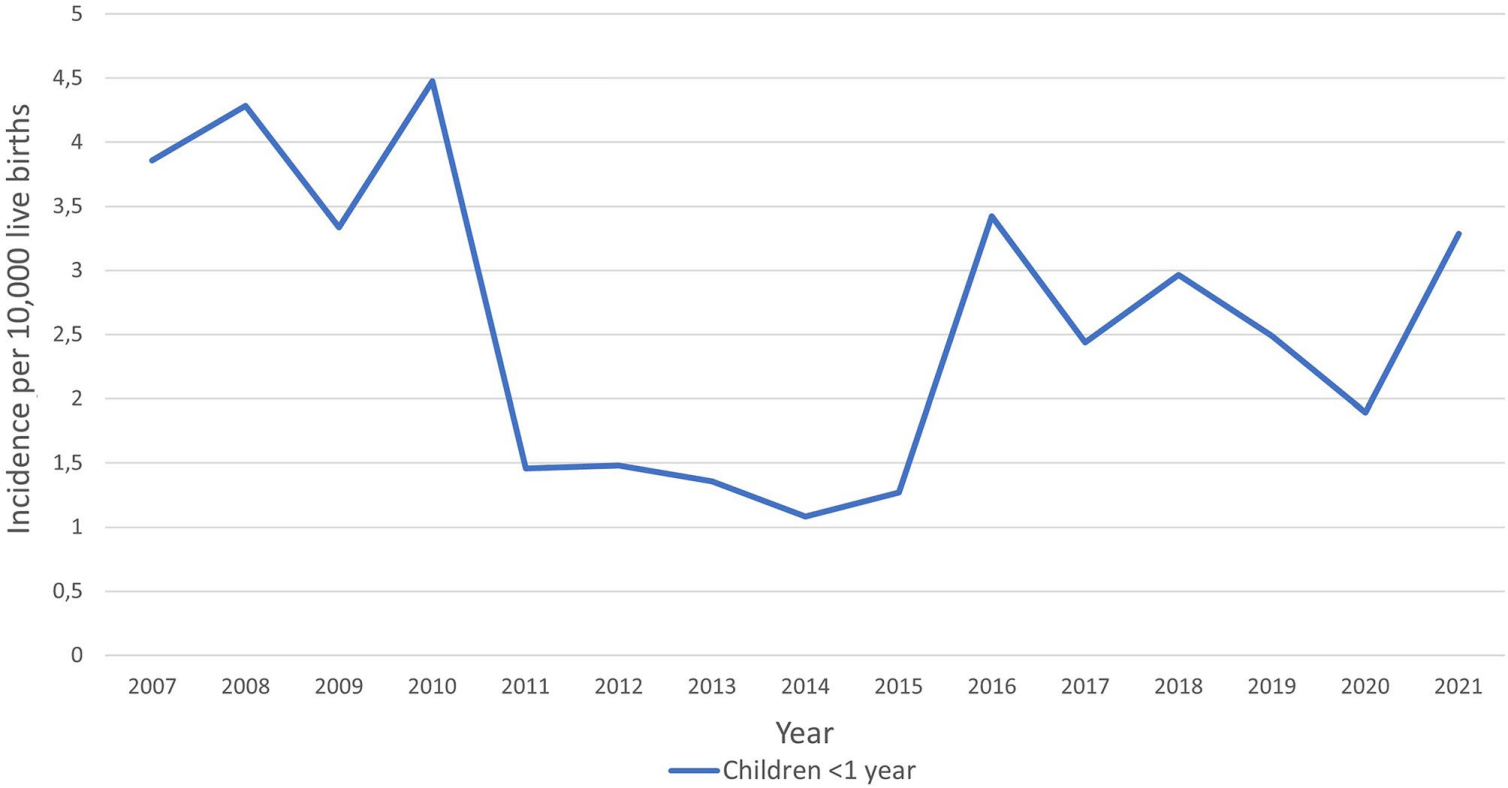
Incidence trends of CT in Poland, 2007–2021.

**Figure 3:**
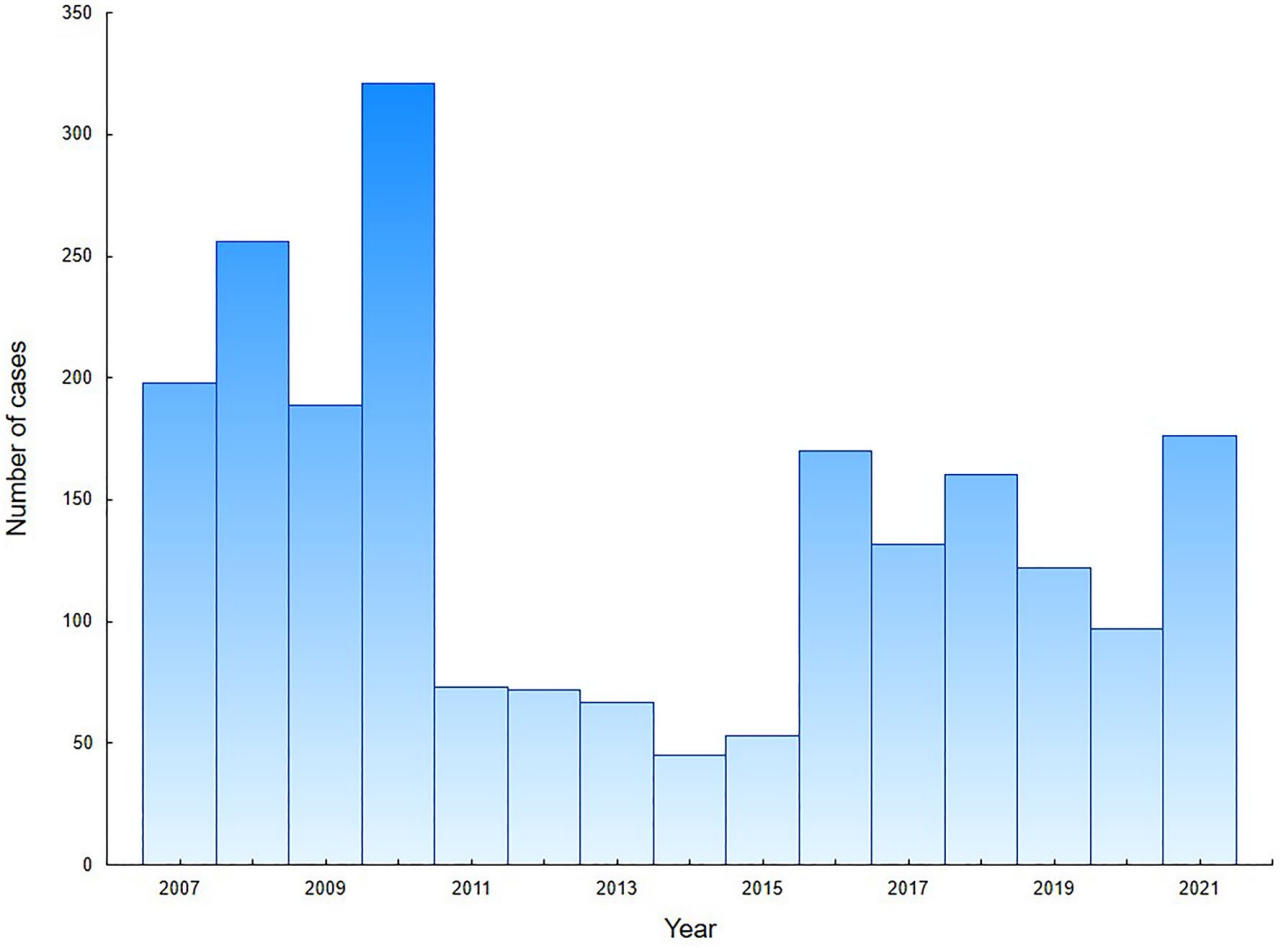
Number of first-time CT hospitalizations in Poland, 2007–2021.

We analysed the total number of 2131 hospital discharge records of CT patients in 2007–2021. The study group consisted of 1504 hospitalization records of first-time diagnosis of CT in newborns: 763 males (50.7%) and 741 females (49.3%). In this group, 89.7% patients were infants up to 90 days of age and 69.8% were neonates up to 28 days of age. The distribution of age at the diagnosis during first-time hospitalizations is presented in Fig. 1. The mean and median age was 31 days (95% CI 29–34 days) and 10 days (IQR: 0–36 days), respectively. Based on hospital registry, the mean annual CT incidence was estimated to be 2.6 per 10,000 live births (95% CI 2.0–3.2 per 10,000 live births). The incidence of CT fluctuated between 2007 and 2021, with the highest incidence in 2010 and the lowest in 2014. In Fig. 2, we presented the incidence of CT in Poland, after a comparison with public demographic data. The incidence was expressed as the number of cases per 10,000 live births per year. No significant linear trend was found considering the entire period studied. To verify significant changes in CT incidence during the study period, the incidence (Fig. 2) was first divided into 3 periods: 2007–10 (A), 2011–15 (B) and 2016–21 (C), and then compared using the Kruskal–Wallis H test (*P* < 0.01). In post-hoc analysis that used multiple comparisons of mean ranks for all groups, there was a significantly higher incidence of CT in period A compared to period B (*P* < 0.005). No significant differences were observed between periods A and C or B and C. Figure 3 shows the number of first-time hospitalizations of infants in Poland with a diagnosis of CT in each year of the analysed period. The extreme values for the data occur at the same time points as presented in Fig. 2. There were no statistically significant differences between the incidence of CT across sex (chi-square test) or in the comparison between rural and urban areas (chi-square test). In the analysed period, 8 deaths were recorded (0.5% of all patients): 5 males and 3 females. In 6 of the 8 deaths, congenital toxoplasmosis was identified as the primary or secondary cause of death. In the remaining cases, cardiovascular diseases were the cause of death in one and respiratory diseases in the second case.

The most common comorbidities were congenital malformations, deformations and chromosomal abnormalities (ICD-10: Q00–Q99; 13.4% of all patients), neonatal jaundice from other and unspecified causes (P59.0–P59.9; 11.8%), congenital cytomegalovirus infection (P35.1; 11.2%), disorders related to short gestation and low birth weight, not elsewhere classified (P07.0–P07.9; 5.9%), certain infectious and parasitic diseases (A00–B99; 5.4%) and diseases of the nervous system (G00–G99; 5.2%).

## Discussion

As shown in Fig. 1, more than two-thirds of the study group were in the neonatal group of patients aged until 28 days. The median age was 10 days, while the average age was 31 days. The age distribution may be due to the diagnostic capabilities of CT, including prenatal serological screening for toxoplasmosis, which is imposed by legal regulations in Poland^24^. In addition, in the group of mothers suspected of having active toxoplasmosis during pregnancy, amniocentesis can be performed to determine *T. gondii* DNA material based on PCR, which has specificity of 100% and sensitivity of 90%^31^. Furthermore, tests such as prenatal and postnatal ultrasound can highlight characteristic lesions such as calcifications in the brain^10^. Modern diagnostic methods result in the early detection of the disease and the possibility of quick implementation of necessary treatment. CT diagnosis can also take place in the following months of life, due to the existence of a spectrum of symptom severity, depending on the stage of pregnancy at which the foetus was infected^3,4^.

Between 2007 and 2021, there was an average of about 380,000 live births per year in Poland. The incidence of CT in the years 2007–2021 was 2.6 per 10,000 live births. Compared to the previously available data on the Polish population, this is a relatively low rate. Studies from the Poznan region that focused on the years 1998–2000 estimated the incidence of CT at 10.8 per 10,000. Based on the studies conducted in Lodz in 2004–2012 which assessed the serological status of pregnant women, the extrapolation of data set the incidence of CT at 18 per 10,000. None of the studies assessed the incidence of CT at a nationwide level. An additional factor overestimating the rates may have been the fact that the studies were limited to referral centres, which may have aggregated cases of complicated pregnancies, such as those with coexisting toxoplasmosis. In addition, only the first study assessed CT directly, but it was done in the last century, while the second study only extrapolated the serological results of pregnant women to the expected incidence of CT among newborns^26,27^.

A comparison on a global scale indicates that the incidence of the disease in Poland is lower than the assumed global average of 15 cases per 10,000 live births^15^. An analysis of detailed data from individual countries shows that the incidence of CT at 2.6 per 10,000 live births is similar to that in other European countries, such as Austria (1 per 10,000), Denmark (2.1 per 10,000), and France (2.9 per 10,000)^16–18^. These results are also similar to available data from the US (1 per 10,000)^21^. It should be noted that data from the physician-reported case registry in Poland indicate a lower incidence rate than it has been estimated in this study^32^. The official European Centre for Disease Prevention and Control (ECDC) report on CT in 2019 indicates the incidence of 0.51 per 10,000 live births in Europe. When comparing these data with available studies from individual countries, including the study presented here, the information provided by the ECDC may be underestimated, for example, due to international reporting issues^33^. There is some contrast between these results and data from countries such as Morocco (3.9–8 per 10,000), Brazil (4–23 per 10,000), and Panama (18 per 10,000), where the incidence is significantly higher^7,19–21,23^. Differences in CT incidence across regions may be due to factors such as access to medical care, socioeconomic conditions, level of public awareness of TORCH infections, national public health standards, and hygienic conditions. The data for Poland puts this country among other developed Western countries, which may be due to both broad access to modern diagnostic and treatment methods, educational programs and legal solutions to reduce CT infections^24^. The results of a study from China on the causes of infant hospitalizations in the years 2015–2020 showed a very low incidence of CT, as only 0.07 per 10,000 hospitalized children under 1 year of age (5 cases) were reported. These data determined the results only for a selected group of tertiary hospitals in China^22^.

As presented in Fig. 2, the incidence of CT in Poland in the years 2007–2021 fluctuated, with the highest incidence in 2010 and the lowest one in 2014. As we can observe in Fig. 2, lower incidence values were registered between 2011 and 2015, relative to other years of the study period. Globally, a decreasing trend in seropositivity against *T. gondii* is observed, but this does not always reflect a direct decrease in CT incidence. It is believed that the influence of a number of factors, such as demographic structure, cyclical changes in the percentage of the population susceptible to infection, and dynamically changing legal and socio-health conditions may result in a sinusoidal trend in CT incidence in individual societies over many years^34^.

The study analysed comorbidities. In the study group, the most common concomitant diagnoses were those described in the literature as occurring in the course of CT, such as malformations and deformations (13. 3% of all patients), neonatal jaundice (11.9%), prematurity and low birth weight (5.8%) and diseases of the nervous system (5.2%). The above symptoms and diseases correspond to those typically seen in CT. The most characteristic triad of symptoms are chorioretinitis, intracranial calcifications, and hydrocephalus, but prematurity, spontaneous abortion, stillbirth, liver or spleen enlargement, jaundice, fever, microcephaly, hearing abnormalities, pneumonia, myocarditis, and many others can also be observed^2,8–10^. Attention is also drawn to the frequent congenital co-infection with CMV (11.3%), which could have exacerbated the neurological changes caused by both pathogens.

During the study, 8 hospital deaths of CT patients occurred (0.5% of the whole group)—congenital toxoplasmosis was identified as the primary or secondary cause of death in 6 of the 8 deaths. In the remaining cases, cardiovascular diseases were the cause of death in one and respiratory diseases in the second case. Given the possibility of developmental abnormalities of various organs and various severity in CT, these deaths may also have been indirectly related to CT^3,4^. This is a relatively low figure. Another study conducted in the years 1974–2007, where causes of infant deaths in Japan were analysed, found two neonatal deaths due to CT (0.001% of all neonatal deaths)^35^. However, the mortality rate may be underestimated due to miscarriages early in pregnancy, which can occur during intrauterine infection.

No significant gender differences were observed in the study with regard to the incidence, which is consistent with the ECDC report on CT in Europe, where the female-to-male ratio was 1:1^33^.

Based on data for Poland from 2007 to 2021, there were no significant differences between the incidence of CT according to the place of residence (urban versus rural areas). Although based on data from a single centre in the capital city, rural residence was found to be an independent risk factor for toxoplasmosis in pregnant women in Poland, this does not translate into a higher incidence of CT in infants from rural areas^25^. The lack of differences between urban and rural areas may also be due to the standards of care applied throughout the country, which are defined by legal regulations. These standards specify, in addition to the methods of early detection and implementation of effective treatment, appropriate education of pregnant patients on the potential risks and ways of infection with *T. gondii*^24^. Improvement of access to medical care in Poland, as well as availability of educational materials and recommendations on the Internet may lead to a reduction in the differences between incidence in urban and rural populations, despite greater environmental exposure.

Despite its advantages, the presented study also has its limitations, mainly due to its retrospective nature. In the course of the analysis, we did not verify the findings on the basis of which CT was diagnosed. We assume that the diagnosis is based on the most current, widely used diagnostic criteria for this disease entity. The incidence of CT in Poland was assessed on the basis of first-time hospitalizations for the disease, due to the need for specialized testing and implementation of treatment when the disease occurs in newborns. In addition, the date of hospitalization after birth may inaccurately represent the actual date of diagnosis, due to the possibility of prenatal detection. Due to these limitations, the incidence by year may have been represented inaccurately, but the long observation period and large sample size of the data obtained from the national registry may minimize this imprecision.

## Conclusions

The average incidence of CT in Poland in 2007–2021 is similar to that in other European countries. The periodic increase in CT incidence indicates the need for further education of the population and the development of effective prenatal diagnosis and treatment programs. In our study, hospital mortality among infants with CT in Poland was relatively low. Despite potentially increased environmental exposure, no significant differences in CT incidence were found between rural and urban areas. Periodic fluctuations in the number of cases of congenital toxoplasmosis indicates the need to develop effective prevention programs to effectively counteract the disease and its consequences.

## Data Availability

The data that support the findings of this study are available on request from the corresponding author.
