# Author Correction: Congenital toxoplasmosis among hospitalized infants in Poland in the years 2007–2021: study based on the national hospital registry

**DOI:** 10.1038/s41598-023-41563-x

**Published:** 2023-08-31

**Authors:** Michał Rząd, Krzysztof Kanecki, Katarzyna Lewtak, Paweł Goryński, Piotr Tyszko, Izabela Lewandowska-Andruszuk, Aneta Nitsch‑Osuch

**Affiliations:** 1 Department of Social Medicine and Public Health, Medical University of Warsaw, 3 Oczki Str., 02-007 Warsaw, Poland; 2 Doctoral School, Medical University of Warsaw, Warsaw, Poland; 3 National Institute of Public Health NIH - National Research Institute, Warsaw, Poland; 4 Institute of Rural Health in Lublin, Lublin, Poland; 5 Department of Obstetrics, Gynaecology and Gynaecologic Oncology, Mazovian Specialist Hospital, Radom, Poland; 6 Faculty of Medical Sciences and Health Sciences, Kazimierz Pulaski University of Technology and Humanities, Radom, Poland

Correction to: *Scientific Reports* https://doi.org/10.1038/s41598-023-38270-y, published online 08 July 2023

The original version of this Article contained errors in the names of the authors Michał Rząd, Krzysztof Kanecki, Katarzyna Lewtak, Paweł Goryński, Piotr Tyszko, Izabela Lewandowska-Andruszuk & Aneta Nitsch‑Osuch, which were incorrectly given as Rząd Michał, Kanecki Krzysztof, Lewtak Katarzyna, Goryński Paweł, Tyszko Piotr, Lewandowska‑Andruszuk Izabela & Nitsch‑Osuch Aneta.

The original Article has been corrected.

